# Translation and Validation of the Hearing Environments and Reflection on Quality of Life (HEAR-QL) Questionnaire for Children and Adolescents in Arabic

**DOI:** 10.7759/cureus.38936

**Published:** 2023-05-12

**Authors:** Maha W AlNowaiser, Reem M Bakraa, Malak M Alamoudi, Razan A Basonbul, Afnan F Bukhari, Faisal Zawawi

**Affiliations:** 1 Otolaryngology - Head and Neck Surgery, King Abdulaziz University, Jeddah, SAU; 2 Emergency, King Abdulaziz University, Jeddah, SAU

**Keywords:** arabic version, back translation, children, quality of life, hearing loss

## Abstract

Background

There are numerous quality-of-life (QoL) assessment tools available; however, only a few are designed specifically for children with chronic conditions. Among these assessment tools are the Hearing Environments and Reflection on QoL questionnaires for children (HEAR-QL26, HEAR-Q28) developed by Washington University. Unfortunately, there are no other tools that assess hearing loss, and none of them are in Arabic. This paper aims to adapt the HEAR-QL to Arabic and provide an accessible method of measuring the QoL of children with hearing loss in our Arabic-speaking populations.

Methodology

An independent medical translator translated the HEAR-QL26 and HEAR-QL28 into Arabic. The translations were then examined by two bilingual, native Arabic-speaking otolaryngologists who modified the inadequate questions. Back-translation of the Arabic version into English was subsequently performed by an independent translator. Intra-rater reliability was tested for each of HEAR-QL26 and HEAR-QL28 using 10 participants for each survey, where the participants answered the surveys twice with a period of two weeks between them. A pilot study was conducted which had a total of 40 participants divided equally between the two surveys where each group had an equal number of hearing participants and participants with hearing loss.

Results

Both HEAR-QL26 and HEAR-QL28 were validated with an overall intra-rater reliability of 88.85% and 87.86% respectively. In the pilot study, the HEAR-QL26 participants with normal hearing scored a median of 2437.5, while the participants with hearing loss scored a median of 1837.5 (p = 0.001). Moreover, HEAR-QL28 participants had a median score of 2725 among participants with normal hearing and 1725 for participants with hearing loss (p = 0.001).

Conclusion

HEAR-QL is a well-established QoL in children with hearing loss. The validated Arabic adaptation can now be used to measure deafness in Arabic-speaking children.

## Introduction

Hearing loss occurs when there is a disturbance in the usual functioning of any part of the ear. This includes the following: the outer ear, middle ear, inner ear, acoustic nerve, and auditory system [1]. The World Health Organization (WHO) estimates that over 6% (466 million people) of the world’s population have disabling hearing loss; 34 million of them are children [2-3]. The prevalence of hearing loss among children in Saudi Arabia was estimated to be 13% [4]. A screening program was launched in 2016 by the Saudi Ministry of Health for the detection of hearing loss among newborns [5]. The earlier the occurrence of hearing loss in the child's life, the more likely the impact on the child's development would be. Likewise, if the hearing loss was detected earlier, a better outcome and impact on the quality of life would be observed [6-7]. Early intervention by hearing assistive devices allow children with hearing loss the opportunity to improve their quality of life [8-9].

Many quality-of-life assessment tools are available, such as the Pediatric Quality of Life (PedsQL); however, few are specific to children with chronic conditions. Among these assessment tools are the Hearing Environments And Reflection on Quality of Life Measurement for Children surveys (HEAR-QL26, HEAR-Q28) developed by Washington University. These surveys assess the quality of life among children with hearing loss by examining many factors, such as environments, activities, and feelings using the HEAR-QL26 and hearing situations, social interactions, school difficulties, and feelings using the HEAR-QL28. HEAR-QL is reportedly more sensitive and effective than PedsQL [10]. Unfortunately, there are no other tools that assess hearing loss and none of them are in Arabic. HEAR-QL has undergone translation and cultural adaptation, to be used outside its original environment. Often studies rely on back-translation since research demonstrates that comparing the primary source language version with the back-translated version of texts helps confirm the accuracy of the translation. This has been the most widely used method of "ensuring" the similarity between the original and the target text [11]. Moreover, it is by far the most popular assessment tool used in international and cross-sectional research. It is important to mention that back-translation has never been used as a standalone method; it is part of a complicated process of translation that differs based on the field of research and the purposes of the study [12]. This paper aims to adapt the HEAR-QL to Arabic and provide an accessible method of measuring the quality of life of children with hearing loss in our Arabic-speaking population.

## Materials and methods

Development of the Arabic versions of HEAR-QL

After obtaining permission from Washington University and the original authors of the English version of HEAR-QL, an independent medical translator translated the HEAR-QL26 and HEAR-QL28 into Arabic. The translations were then examined by two bilingual native Arabic-speaking otolaryngologists who modified the inadequate questions. Back-translation of the Arabic version into English was subsequently performed by an independent translator who was not aware of the English versions of the HEAR-QL. The source of measurement used in this study is described in a paper by Umansky et al. (2011) [8] and was provided to the authors with permission.

Validity of the Arabic questionnaire

The study was approved by the institutional ethics review board. Written and informed consent was obtained from all participants prior to enrollment in this study.

In this study, two groups were established; Group A consisted of 10 children between the ages of 7-12 years, while Group B consisted of 10 children between the ages of 13-18 years. The HEAR-QL26 was given to Group A, while the HEAR-QL28 was given to Group B. All the children involved in this study had normal hearing, had Arabic as their first language, and were not suffering from any cognitive impairment. 

Pilot study

The pilot study included 40 children, half of whom participated in the HEAR-QL26 and the other 20 in the HEAR-QL28 survey. Among the 20 participants who completed the HEAR-QL26 included 10 participants with normal hearing and 10 participants with hearing loss. The HEAR-QL28 participants were also divided into groups of 10 participants with normal hearing and 10 participants with hearing loss. A comparison was also made between the participants with normal hearing and hearing loss in both surveys.

Statistical analysis

Both the HEAR-QL26 and HEAR-QL28 use a brief scoring system comprising a 5-point scale. The options were: almost always (0), often (1), sometimes (2), almost never (3), and never (4). The scores for the options were converted to 0-100 scores distributed as follows: 0=0, 1=25, 2=50, 3=75, and 4=100. With respect to hearing, lower scores indicated a poorer quality of life, while higher scores indicated a greater quality of life.

## Results

HEAR-QL26 and HEAR-QL28

A total of 20 participants with normal hearing, 10 for each survey, were asked to join this study and answer the translated HEAR-QL26 and HEAR-QL28 twice separated by a period of two weeks to determine the validity of the translated questionnaire.

The HEAR-QL26 participants included 10 children between the ages of 7 and 12. The median score of the participants was 2462.5 (94.7%) in the first round, whereas, they scored a median of 2575 (99.04%) in the second round.

Another 10 participants between the ages of 13 and 18 answered the HEAR-QL28 translated survey. On the first attempt, the participants had a median score of 2687.5 (95,66%); upon taking the survey two weeks later, they showed a median score of 2737.5 (97.8%).

Intra-rater reliability

The HEAR-QL26 survey was validated with overall intra-rater reliability of 88.85%, while the HEAR-QL28 survey was validated with 87.86% overall intra-rater reliability. 

Pilot study

The pilot study included 40 children, half of which took the HEAR-QL26 and the other half took the HEAR-QL28 survey. Among the 20 participants that took the HEAR-QL26, 10 were participants with normal hearing and 10 were participants with hearing loss, both with a median age of 10 years. The participants with normal hearing scored a median of 2437.5 (93.75%), while the participants with hearing loss scored a median of 1837.5 (70.675%) (p = 0.001) (Tables 1-2). 

**Table 1:** HEAR-QL26 results of children younger than 13 years with normal hearing This table demonstrates the HEAR-QL26 results for participants with normal hearing.

Participant	Gender	Age	HEAR QL 26 score	HEAR QL 26 score (out of 100)
1	M	7	2600	100
2	F	9	2600	100
3	F	10	2450	94.23
4	F	11	1875	72.11
5	M	12	2425	93.27
6	M	11	2450	94.23
7	M	10	2500	96.15
8	F	9	2400	92.3
9	M	10	2350	90.4
10	M	11	2375	91.35

**Table 2:** HEAR-QL26 results of children younger than 13 years with hearing loss This table demonstrates the HEAR-QL26 results for participants with hearing loss

Participant	Gender	Age	HEAR QL 26 Score	HEAR QL 26 score (out of 100)
1	F	8	1950	75
2	F	7	2225	85.58
3	M	10	2100	80.77
4	F	9	1300	50
5	M	11	1700	65.38
6	M	8	2000	76.9
7	F	11	1800	69.23
8	M	10	1500	57.69
9	F	12	1400	53.85
10	F	10	1875	72.12

The HEAR-QL28 participants were similarly divided into 10 participants with normal hearing and 10 participants with hearing loss with a median age of 15 and 15.5 years respectively, the median score was 2725 (97.3%) for the participants with normal hearing and 1725 (61.61%) for the participants with hearing loss (p = 0.001). Tables 3-4 provide further details regarding each of the participants’ age, sex, and HEAR-QL28 score.

**Table 3:** HEAR-QL28 results of children 13 years and older with normal hearing This table demonstrates the HEAR-QL28 results for participants with normal hearing

Participant	Gender	Age	HEAR QL 28	HEAR QL 28 (out 100)
1	F	15	2800	100
2	F	16	2675	95.5
3	F	15	2650	94.64
4	M	13	2700	96.4
5	F	14	2800	100
6	M	17	2600	92.85
7	M	16	2750	98.21
8	M	15	2675	95.5
9	M	15	2775	99.1
10	F	17	2750	98.2

**Table 4:** HEAR-QL28 results of children with hearing loss aged 13 years and older This table demonstrates the HEAR-QL28 results for participants with hearing loss

Participant	Gender	Age	HEAR QL 28 Score	HEAR QL 28 (out 100)
1	M	13	1650	58.93
2	M	15	1800	64.29
3	F	15	1525	54.46
4	F	17	1875	66.97
5	F	16	2125	75.89
6	F	17	2325	83
7	F	17	1025	36.6
8	M	15	1200	42.86
9	F	14	1650	58.93
10	M	16	2425	86.6

## Discussion

Translating the surveys HEAR-QL26 and HEAR-QL28 from English to Arabic was of great importance since it has provided a validated tool to assess the quality of life in Arabic-speaking children with hearing loss. Each survey scored an intra-rater reliability of over 85%; using the Arabic version of the survey could enable further research into the quality of life of Arabic-speaking children with hearing loss. 

Hearing loss clearly puts a person at a disadvantage, and even if it is obvious that this places children with hearing impairment in vulnerable positions, the actual impact on quality of life still requires to be defined [13]. One of the most efficient measuring tools for assessing the quality of life is the HEAR-QL, therefore translation of this assessment tool to Arabic is extremely crucial. Arabic is the official language of 22 countries; Arabic speakers account for more than 300 million speakers in the world [14-15]. This paper is one of the few studies that assess the quality of life based on hearing loss. We used the HEAR-QL26 and HEAR-QL28 questionnaire, which includes the assessments of the hearing environment with social interaction, continued activities, feelings, and school difficulties. The WHO defines the quality of life as encompassing six domains including the physical domain, psychological domain, level of independence, social relationships, environment, and spirituality/religion/personal beliefs [16].

Hearing impairment has significant impacts on those who are affected, regardless of their age or degree of disability. Owing to a lack of early auditory stimulation and spoken communication, children and youth who were born with hearing impairment or acquired hearing impairment at a younger age are at risk of delayed speech, language, cognitive, and social development [17]. Initial delays can lead to decreased social interaction, poor academic achievement, feelings of isolation, and low self-esteem, all of which can result in increased behavioral, socio-emotional, or learning challenges. These challenges may persist into adolescence and adulthood [18]. Thus, hearing loss is a life-changing occurrence that affects all the elements and phases of a person's life. Supporting children and young people with hearing loss necessitates flexible health and educational services that can be adjusted based on their changing requirements [19]. By assessing their quality of life, the needs of the children and young people could be attended.

Many studies have demonstrated that children with hearing loss had lower scores on quality-of-life questionnaires compared to their peers with normal hearing [10,20]. Similarly, our pilot study shows significantly higher scores in participants with normal hearing than those in participants with hearing loss in each of HEAR-QL26 and HEAR-QL28. On the other hand, a previously published study described a lack of difference in the quality of life in children with hearing impairment as compared to those with normal hearing [21].

There is a significant relationship between hearing rehabilitation and quality of life; specifically, the students who received hearing rehabilitation reported a higher quality of life than those who never received hearing rehabilitation. However, the majority of children with hearing impairment do not wear hearing aid devices owing to economic and stigmatic reasons, especially in middle- and low-income countries [22]. For that reason, the presence of a QoL measuring tool that is validated to the patients' language would help study the outcome of children with various disorders, and in this case, hearing loss.

## Conclusions

HEAR-QL is a well-recognized tool to measure the quality of life in children with hearing loss, and in this Arabic adaptation of the surveys, we provide a validated tool measuring the quality of life in children with hearing loss who speak Arabic as a native language.
